# HIV infection, hunger, breastfeeding self-efficacy, and depressive symptoms are associated with exclusive breastfeeding to six months among women in western Kenya: a longitudinal observational study

**DOI:** 10.1186/s13006-019-0251-8

**Published:** 2020-01-16

**Authors:** Emily L. Tuthill, Joshua D. Miller, Shalean M. Collins, Elizabeth M. Widen, Maricianah Onono, Sera L. Young

**Affiliations:** 1 Department of Community Health Systems, School of Nursing, University of California, San Francisco, San Francisco, CA USA; 2 Department of Anthropology, Northwestern University, Evanston, IL USA; 3 Department of Nutritional Sciences, College of Natural Sciences, University of Texas at Austin, Austin, TX USA; 4 Center for Microbiology Research, Kenya Medical Research Institute, Nairobi, Kenya

**Keywords:** Breastfeeding determinants, Exclusive breastfeeding, Food insecurity, Perinatal depression, HIV, Breastfeeding self-efficacy, Hunger, Sub-Saharan Africa

## Abstract

**Background:**

Exclusive breastfeeding for the first six months of life is recommended for all infants. However, breastfeeding rates remain suboptimal; around 37% of infants are exclusively breastfed for the first six months globally. In Nyanza region, western Kenya, numerous challenges to breastfeeding have been identified, including food insecurity, hunger, depressive symptoms, and HIV infection. Yet, evidence to inform our understanding of how these problems influence women’s breastfeeding behaviors across time is lacking. We therefore sought to examine these factors and how they interact to affect the initiation and duration of exclusive breastfeeding in this region. We hypothesized that women experiencing greater food insecurity, hunger, and/or depressive symptoms would be less likely to maintain exclusive breastfeeding for six months than women who were food secure or not depressed. We also hypothesized that women living with HIV would be more likely to maintain exclusive breastfeeding to six months compared to HIV-uninfected women.

**Methods:**

Women in Pith Moromo, a longitudinal cohort study in western Kenya, were surveyed at two antenatal and three postpartum timepoints (*n* = 275). Data were collected on breastfeeding behavior and self-efficacy, maternal food insecurity and hunger, maternal psychosocial health, and HIV status. Cox proportional hazards models were used to identify predictors of early exclusive breastfeeding cessation.

**Results:**

The majority of women (52.3%) exclusively breastfed for the first six months. In the final multivariable Cox proportional hazards model, living with HIV was associated with a 64% decrease in the rate of early exclusive breastfeeding cessation. Additionally, the rate of early exclusive breastfeeding cessation increased by 100 and 98% for those experiencing probable depression or hunger, respectively. Although there was no main effect of breastfeeding self-efficacy, the interaction between breastfeeding self-efficacy and hunger was significant, such that the rate of early exclusive breastfeeding cessation was predicted to decrease by 2% for every point increase in breastfeeding self-efficacy score (range: 0–56).

**Conclusions:**

This study contributes to previous work demonstrating that women living with HIV more consistently exclusively breastfeed and suggests that rates of exclusive breastfeeding could be increased through targeted support that promotes maternal mental health and breastfeeding self-efficacy, while reducing maternal hunger.

**Trial registration:**

**Study registration** NCT02974972.

## Background

Despite global recommendations promoting exclusive breastfeeding, and the known benefits of breast milk, rates remain suboptimal. For example, only 37% of infants younger than six months of age are exclusively breastfed in low- and middle-income countries [1]. Known barriers to exclusive breastfeeding range from cultural norms to structural challenges [2–4]. Three factors that plausibly limit exclusive breastfeeding in low-resource settings, however, remain understudied and include food insecurity, poor psychosocial health, and HIV status [5–8].

Many women in western Kenya acutely experience these highly prevalent factors simultaneously [9–11] and their potential interactions may further impede the initiation or maintenance of exclusive breastfeeding [5]. Food insecurity is high in western Kenya, with 10–20% of households experiencing poor or borderline food consumption throughout the year, regardless of seasonal changes or droughts that may exacerbate the experience of food insecurity [9]. A 2013 assessment also found that over 10% of adults had at least one common mental disorder, such as anxiety and depression [11]. Additionally, the prevalence of HIV infection among women is high at 17.7%, with the highest prevalence among childbearing women [10]. These modifiable risk factors, what mediates them, and how they interact to influence breastfeeding behaviors, have not been well described.

Pregnant and postpartum women are uniquely vulnerable to food insecurity and hunger, both of which can affect breastfeeding via multiple, and sometimes overlapping, pathways. During pregnancy and postpartum, women experience higher nutrient demands, physical limitations that make procuring and preparing food difficult, as well as constraints related to resource accessibility, including employment [12, 13]. Food procurement can separate breastfeeding mothers from their infants [14], and increased maternal stress related to food insecurity can cause decreased milk production and limit breastmilk intake [15–17].

Evidence also suggests that food-insecure women are more likely to believe that supplemental feeding is necessary because they feel unable to provide adequate nutrition to their infant through exclusive breastfeeding [18, 19]. Furthermore, inadequate resources to pay for transportation or the opportunity costs of clinic visits [14] may impede women from attending their clinic appointments, where infant feeding counseling may bolster breastfeeding self-efficacy. Conversely, food insecurity has been shown to facilitate exclusive breastfeeding, when, regardless of intentions, women have no other foodstuffs available to provide to their infants [20]. As such, food insecurity has been shown to have varying impacts on breastfeeding behaviors across different contexts [20–22].

Depression is associated with both food insecurity and HIV [23–27], and contributes to negative health outcomes [28, 29], including disrupting breastfeeding behaviors [30, 31]. Prevalence of depression among pregnant and postpartum women is high globally, ranging from 10 to 47% [7, 32, 33]. Depressive symptoms can manifest as difficulty bonding with the newborn, severe fatigue, cognitive problems, thoughts of harming the newborn or self, and reduced interest in daily activities [34]. These symptoms can result in numerous negative health outcomes, including disruption of parenting behavior [31], poor attachment [35, 36], and lower rates of breastfeeding [30, 37]. These, in turn, can result in long-term health complications for infants, including impaired child growth and delays in cognitive, emotional, and behavioral development [38–40].

Compared with women who are HIV-uninfected, women living with HIV face distinct barriers to exclusive breastfeeding, such as disease-related stigma [41] and the management of a chronic illness requiring daily medication. The World Health Organization’s (WHO) recommended strategy for preventing the transmission of HIV from mother-to-child includes exclusive breastfeeding (in addition to lifelong antiretroviral therapy) [42, 43], making this practice of even greater consequence for women living with HIV. These recommendations have been integrated into standard of care and the education delivered at many antenatal clinics in Kenya [44], including the Family AIDS Care & Education Services (FACES) perinatal clinics where our study was conducted. It is possible that such messages and emphasis in clinical settings could lead to increases in exclusive breastfeeding among women living with HIV.

Using longitudinal data from pregnant and postpartum women in western Kenya, we investigated understudied breastfeeding predictors. We hypothesized that women experiencing greater food insecurity, hunger, and/or depressive symptoms would be less likely to maintain exclusive breastfeeding for six months than women who were food secure or not depressed. Given the increased efforts to promote exclusive breastfeeding through the prevention of mother-to-child transmission of HIV programs, we also hypothesized that women living with HIV would be more likely to maintain exclusive breastfeeding to six months compared to HIV-uninfected women.

## Methods

### Study setting and population

Data were drawn from Pith Moromo, a longitudinal observational cohort study designed to explore the consequences of food insecurity and HIV during the first 1000 days (NCT02974972). Pregnant women (*n* = 371) were recruited from seven rural, peri-urban, and urban FACES antenatal clinics in Nyanza region, Kenya (near Lake Victoria) between September 2014 and June 2015. Women were eligible for inclusion if they were within their first seven months of pregnancy (assessed using last menstrual period on antenatal cards) and intended to live in the catchment area until their infant(s) reached at least nine months of age. All women living with HIV were prescribed antiretroviral therapy per national guidelines. Quota sampling was used in order to achieve equal numbers of pregnant HIV-unifected women and pregnant women living with HIV (confirmed using colloidal gold rapid tests) by food insecurity categories, assessed using the nine-item Individual Food Insecurity Access Scale (low 0–9, moderate 10–18, and severe 19–27) [45]. Women with HIV were oversampled to detect differences in primary study outcomes (e.g. maternal BMI) by HIV status at a power of 0.8. Luo is both the predominant language spoken and the ethnic group with which the majority of individuals identify.

### Data collection

Survey data were collected by Kenyan clinic-based study nurses using paper forms and tablet-based electronic surveys. Interviews were conducted at five time points: twice during the index pregnancy (16–30 weeks and 24–40 weeks) and three times after delivery (1.5, 3, and 9 months postpartum). Sociodemographic characteristics, including age, religion, and ethnic group, were collected at baseline. A principal component analysis was performed on reported household assets and used to represent household wealth. Maternal and infant weight and height/length were collected at all visits.

Women were queried about knowledge and intention to breastfeed at the second antenatal visit. Of note, women received standard-care counseling on breastfeeding during their antenatal appointments, with no additional counseling from participation in the study. Women were considered knowledgeable about breastfeeding if they responded that infants should be exclusively breastfed for six months and did not indicate that any other foods or fluids, aside from medicines, could be given. Women were also asked if they intended to exclusively breastfeed for the first six months after birth.

At the first postnatal visit, mothers reported on breastfeeding self-efficacy (range: 0–56) [46], defined as the confidence one has in their perceived ability to breastfeed [47], and breastfeeding social support (range: 0–18), defined as the interactions that convey caring, trust, and love to the breastfeeding mother, or task and knowledge sharing that directly assist that person [48]. Participants also reported on infant feeding practices at all postnatal visits, including whether other foods or fluids were provided to the infant, and when. Duration of exclusive breastfeeding was operationalized as the number of days between birth (date extracted from child clinic cards) and the introduction of foods or fluids other than human milk, based on maternal recall.

Exclusive breastfeeding was defined using the WHO standard as having provided only breast milk and no other foods or fluids (except medicine). Following reported methodologies, we used both a six-month and 5.5-month cut-off for determining whether infants were exclusively breastfed or not [49–51].

Maternal dietary diversity was assessed at each time point using a 24-h food frequency questionnaire [52]. Maternal food insecurity was measured using the Individual Food Insecurity Access Scale (range: 0–27) [45], which asks about experiences of food insecurity in the past four weeks. Maternal hunger is a measure of severe food insecurity derived from responses to the three most extreme experiences queried in the Individual Food Insecurity Access Scale (range: 0–6) [53]. Individuals were then classified as having low (0–1), moderate (2–3), or high (4–6) hunger.

Finally, maternal perceived stress and depression were measured at 1.5 and 9 months postpartum. Depression was assessed using the Center for Epidemiologic Studies-Depression Scale (CES-D, range: 0–60) [54]. We used the cut-off of a score of 17 or higher for probable depression [55, 56]. Maternal stress levels were measured using the Perceived Stress Scale (PSS) (range: 0–40) [57].

### Data analysis

Statistical analyses were conducted using Stata 14.0 software with an α of 0.05. Sociodemographic characteristics were compared between women who exclusively breastfed to six months and women who did not using chi-square and *t*-tests. Cox proportional hazards models were used to identify predictors of early exclusive breastfeeding cessation. In multivariable models, this is a preferred technique because it can account for the length of exclusive breastfeeding for each mother-infant dyad. Time-variant predictors (e.g. food insecurity, depression) measured at the first postnatal visit were used to approximate experiences at delivery. Significant predictors (*p* < 0.2) of exclusive breastfeeding duration in bivariate analyses were included in multivariable Cox proportional hazards models; variables were then eliminated using a backwards stepwise approach (*p* < 0.1).

## Results

### Cohort characteristics

Three hundred and seventy-one pregnant women were enrolled into Pith Moromo and 311 ultimately completed the study; total loss to follow-up was 8.1% (Additional file 1: Figure S1 – Study Flow). Of these 311 participants, complete infant feeding information was available for 275 women.

Using cut-offs of 5.5 and 6 months, 53.5 and 52.3% of women in the study exclusively breastfed, respectively. Given that the prevalence of exclusive breastfeeding did not differ significantly between these two cut-offs, we only report results for exclusive breastfeeding for the first six months of life; results using the 5.5-month cut-off are reported in Additional file 4: Table S1.

Most sociodemographic characteristics were similar between women who exclusively breastfed for six months and those who did not (Table 1), with the exceptions of HIV, BMI, and intention to breastfeed. The majority of participants (86.6%) were Luo. Most participants did not receive secondary education (61.7%) and were multiparous (72.5%).

**Table 1:** Health and sociodemographic characteristics of participants (*n* = 275), by EBF to 6 months

	EBF to 6 months (*n* = 145)	Did not EBF to 6 months (*n* = 130)	*p*-value
Maternal age, mean (sd)	25.5 (4.8)	24.4 (4.8)	0.073
≤ Primary education, %	63.6	59.1	0.461
Married, %	93.1	91.5	0.613
Religion, %			
Catholic	17.1	16.3	0.751
Seventh Day Adventist	23.6	24.8	
Protestant	35.7	31.8	
Other Christian Denomination	20.1	24.0	
African Traditional	3.5	3.1	
Ethnic group, %			
Luo	87.9	85.2	0.517
Household size, mean (sd)	7.1 (3.2)	6.9 (3.4)	0.627
Household wealth, %			
Moderate	23.8	24.6	0.196
High	28.7	38.2	
Household location, %			
Rural	31.7	39.2	0.100
Peri-urban	42.8	15.4	
Urban	25.5	45.4	
HIV-infected, %	62.1	30.8	< 0.001
Primiparous, %	27.8	27.1	0.905
Intention to breastfeed, %	83.8	70.6	0.041
Knowledgeable about breastfeeding, %	4.4	7.6	0.197
Breastfeeding self-efficacy (0–56), mean (sd)	39.0 (0.7)	37.8 (10.4)	0.320
Maternal BMI at 6 weeks postpartum, mean (sd)	22.8 (0.3)	24.0 (0.3)	0.003
Maternal food insecurity at 6 weeks postpartum (0–27), mean (sd)	12.1 (5.4)	11.4 (6.1)	0.296
Maternal hunger at 6 weeks postpartum (0–6), mean (sd)	1.9 (1.3)	1.7 (1.4)	0.201
Social support at 6 weeks postpartum (0–40), mean (sd)	28.6 (7.2)	27.5 (7.7)	0.201
Probable depression (CES-D > 16) at 6 weeks postpartum, %	14.0	22.0	0.093

### Breastfeeding behaviors

At the second antenatal visit, only 6.1% of participants were knowledgeable about exclusive breastfeeding, although most women reported intentions to exclusively breastfeed for at least six months (77.2%). Indeed, the majority of women in this study did initiate breastfeeding (99.0%). Among those who initiated breastfeeding, 50.2% did so within the first hour of birth. The mean length of exclusive breastfeeding was 3.9 (± 2.7) months.

### Predictors of sustained exclusive breastfeeding

A greater proportion of women who intended to exclusively breastfeed for six months ultimately did so (57.3%) relative to those who did not intend to exclusively breastfeed (40.9%). In addition, a significantly greater proportion of women who exclusively breastfed for the first six months were living with HIV (62.1%) compared to women who did not (30.8%) (Table 1).

In the final multivariable Cox proportional hazards model, maternal hunger, but not food insecurity, was also strongly associated with lower rates of exclusive breastfeeding, such that every one-point increase in hunger score was associated with a 98.0% increase in the likelihood of early exclusive breastfeeding cessation. Additionally, the likelihood of early exclusive breastfeeding cessation doubled (HR: 200%) for those experiencing probable depression (Additional file 2: Figure S2 – Survival Curve). Finally, living with HIV was associated with a 64.0% decrease in the likelihood of early exclusive breastfeeding cessation to six months (Table 2; Additional file 3: Figure S3 – Survival Curve).

**Table 2:** Final multivariable Cox proportional hazards model of exclusive breastfeeding to six months among Pith Moromo participants in western Kenya (*n* = 275)

Variable	Adjusted Hazard Ratio	95% Confidence Interval	*p*-value
Maternal hunger score	1.98	(1.03, 3.78)	0.039
Depression (likely vs. none)	2.00	(1.23, 3.23)	0.005
HIV (positive vs. negative)	0.36	(0.23, 0.56)	< 0.001
Breastfeeding self-efficacy	1.01	(0.97, 1.05)	0.583
Maternal hunger score * breastfeeding self-efficacy	0.98	(0.96, 1.00)	0.013
Maternal BMI	1.05	(1.00, 1.11)	0.068

**Fig. 1:**
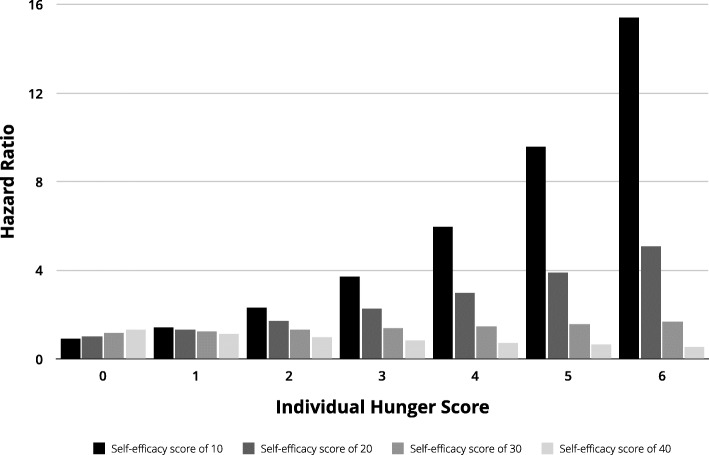
Predicted risk of early breastfeeding cessation by self-efficacy quantile for each individual hunger score. For every 10-point increase in self-efficacy score, there was a consistent, and at some points dramatic, decrease in the hazard ratio for early cessation of exclusive breastfeeding at each level of maternal hunger

Breastfeeding self-efficacy was not significantly associated with exclusive breastfeeding. However, there was a significant interaction between breastfeeding self-efficacy and hunger, such that women with greater self-efficacy across all levels of hunger were more likely to exclusively breastfeed than those with poor self-efficacy (Table 2, Fig. 1). Likewise, individuals who had low breastfeeding self-efficacy experienced a greater increase in the risk of early breastfeeding cessation as hunger increased (Fig. 1). In other words, increased self-efficacy was predicted to buffer against the negative impacts of hunger on exclusive breastfeeding. Holding hunger score constant, the rate of early exclusive breastfeeding cessation was predicted to decrease by 2.0% for every one-point increase in breastfeeding self-efficacy (range: 0–56).

## Discussion

Our first objective was to understand the relationship between food insecurity and exclusive breastfeeding behavior across time among a cohort of women of mixed HIV status in western Kenya. In contrast to our hypothesis, we found no association between food insecurity and duration or prevalence of exclusive breastfeeding at six months. We did, however, find a significant association between greater maternal hunger (a measure of severe food insecurity) and both lower rates of exclusive breastfeeding and early cessation of exclusive breastfeeding (Table 2). Every one-point increase in hunger score was associated with a 98% increase in the rate of early breastfeeding cessation. This is similar to work in Uganda, which found that food-insecure women initiated breastfeeding at the same rate as food-secure women, however, women with greater household food insecurity were less likely to maintain exclusive breastfeeding from four to six months [22]. Similarly, a Canadian study of postpartum women found that food-insecure women initiated breastfeeding at the same rate as food-secure women, although those with greater food insecurity were less likely to exclusively breastfeed to four months [21]. Our study adds to these findings by demonstrating that maternal hunger, a measure of severe food insecurity, is also associated with suboptimal infant feeding outcomes. These findings indicate a critical need to target the most extremely food insecure (also represented by those experiencing the most hunger), as even a one-point increase in hunger score was associated with the cessation of exclusive breastfeeding.

Breastfeeding self-efficacy, although not independently associated with exclusive breastfeeding, had a significant interaction with hunger, such that increased self-efficacy buffered the deleterious impact of hunger on early breastfeeding cessation. Fig. 1 shows that the impact of self-efficacy on the duration of breastfeeding exclusivity is dependent upon maternal hunger level. For example, a 10-point increase in breastfeeding self-efficacy was associated with a 20% decrease in the rate of early breastfeeding cessation. Although previous research has shown self-efficacy to be an important determinant of breastfeeding behavior [46, 58] this is the first study, to our knowledge, that demonstrates its interaction and buffering effect with hunger. Self-efficacy, which reflects one’s confidence in performing a certain behavior [47], likely moderates the effects of hunger by increasing a woman’s confidence in her breastfeeding practices. Mothers with higher self-efficacy may be more responsive to an infant’s feeding cues, have more confidence about the sufficiency of their milk supply, and ultimately maintain exclusive breastfeeding despite being hungry. To increase exclusive breastfeeding, bolstering self-efficacy, especially among women at risk of experiencing hunger, may be a cost-effective approach.

We found support for our second hypothesis that depression would be detrimental for exclusive breastfeeding. In fact, we found that women with depressive symptoms were twice as likely as women without probable depression to stop exclusive breastfeeding before the recommended six-month duration (Table 2). This is consistent with other studies that have reported an association between depression and shorter breastfeeding duration, including in Pakistan [37] and Brazil [59], as well as in a review of studies from 19 countries worldwide [60]. Women with depression are also at higher risk for poor self-efficacy, which may be a pathway through which depression limits exclusive breastfeeding [61].

Regarding our third hypothesis, we found evidence that women living with HIV were more likely to sustain exclusive breastfeeding to six months than HIV-uninfected women (Table 2). There are several possible explanations for the strong and consistent relationship between women living with HIV and exclusive breastfeeding over time, including the integrated and comprehensive prevention of mother-to-child transmission of HIV services provided at the FACES clinics. Since the WHO updated its prevention of mother-to-child transmission of HIV infant feeding guidelines to recommend exclusive breastfeeding to six months [62], there have been major efforts in Kenya at the national and provincial levels to promote exclusive breastfeeding among women living with HIV [44]. In addition, a recent review among postpartum women living with HIV found that a key motivator of exclusive breastfeeding was increased knowledge about the health benefits of breast milk for infants [63]. It should be noted, however, that only 6.1% of women in our cohort were knowledgeable about exclusive breastfeeding at the second antenatal survey, indicating that increased knowledge may not be an influencing factor, or that increased breastfeeding knowledge was acquired after we collected data on this construct. A previous study in this same region of Kenya also found low knowledge of prevention of mother-to-child transmission of HIV, as well as increased rates of exclusive breastfeeding among mothers living with HIV (43.3%) compared to HIV-negative mothers (24.4%) [64]. Thus, more information is needed to explore the quality of information and support women living with HIV are currently receiving, its effectiveness, and how it compares to that received by HIV-uninfected women.

### Limitations

A limitation of the study is that unmeasured determinants may have impacted breastfeeding behavior, such as differences in breastfeeding resources between HIV-uninfected women and women living with HIV. Despite this limitation, our study has several important strengths, including the concurrent examination of many potential predictors of exclusive breastfeeding across time among a cohort of women with a high prevalence of food insecurity and HIV.

## Conclusions

In sum, we found that hunger and probable depression were associated with early exclusive breastfeeding cessation and that women living with HIV were more likely to initiate and maintain exclusive breastfeeding to six months. Additionally, breastfeeding self-efficacy buffered the negative impact of hunger, such that women with greater breastfeeding self-efficacy were predicted to have higher rates of exclusive breastfeeding despite experiencing hunger. These findings highlight the opportunity to adapt current efforts promoting exclusive breastfeeding for *all* infants and their mothers by addressing individual-level (psychological wellbeing, physical health, and nutrition) and structural-level (hunger) determinants of breastfeeding behavior. It may be fruitful to explore the interactive effects of breastfeeding self-efficacy on a range of psychological, disease, and nutritional determinants of breastfeeding. Future research is needed to fully understand how and why women living with HIV are able to exclusively breastfeed for longer durations, as well as if interventions to change modifiable risk factors, such as breastfeeding self-efficacy, are efficacious and effective at scale.

## Supplementary information

**Additional file 1: Figure S1.** PM_EBF_Supp_Figure1.pdf; study flow; Progress of participants through the Pith Moromo study. Three hundred seventy-one women enrolled into the parent study; 8.1% were lost to follow-up.

**Additional file 2: Figure S2.** PM_EBF_Supp_Figure2.pdf; survival curve; Proportion of women (*n* = 275) exclusively breastfeeding through nine months postpartum, by probable depression. The likelihood of early exclusive breastfeeding cessation doubled (HR: 200%) for those experiencing probable depression.

**Additional file 3: Figure S3.** PM_EBF_Supp_Figure3.pdf; survival curve; Proportion of women (n = 275) exclusively breastfeeding through nine months postpartum, by maternal HIV status. Being HIV-positive was associated with a 64.0% decrease in the likelihood of early exclusive breastfeeding cessation to six months.

**Additional file 4: Table S1.** PM_EBF_Supp_Table1.pdf; sensitivity analyses; Bivariate relationship between variables of interest and EBF duration, by cut-off used for determining EBF; only *p*-values reported for exploratory analysis.

## Data Availability

The datasets used and/or analyzed during the current study are available from the corresponding author upon reasonable request.

## Abbreviations

CES-D: Center for Epidemiologic Studies-Depression Scale
FACES: Family AIDS Care & Education Services
HIV: Human Immunodeficiency Virus
PSS: Perceived Stress Scale
WHO: World Health Organization
