# Supplementary material for: HIV infection, hunger, breastfeeding self-efficacy, and depressive symptoms are associated with exclusive breastfeeding to six months among women in western Kenya: a longitudinal observational study
Source: Int Breastfeed J. 2020 Jan 16;15:4. doi: 10.1186/s13006-019-0251-8 (PMC6966845; doi:10.1186/s13006-019-0251-8)
Supplement: Supplementary file 1 — Additional file 1: Figure S1. PM_EBF_Supp_Figure1.pdf; study flow; Progress of participants through the Pith Moromo study. Three hundred seventy-one women enrolled into the parent study; 8.1% were lost to follow-up. [file 13006_2019_251_MOESM1_ESM.pdf]

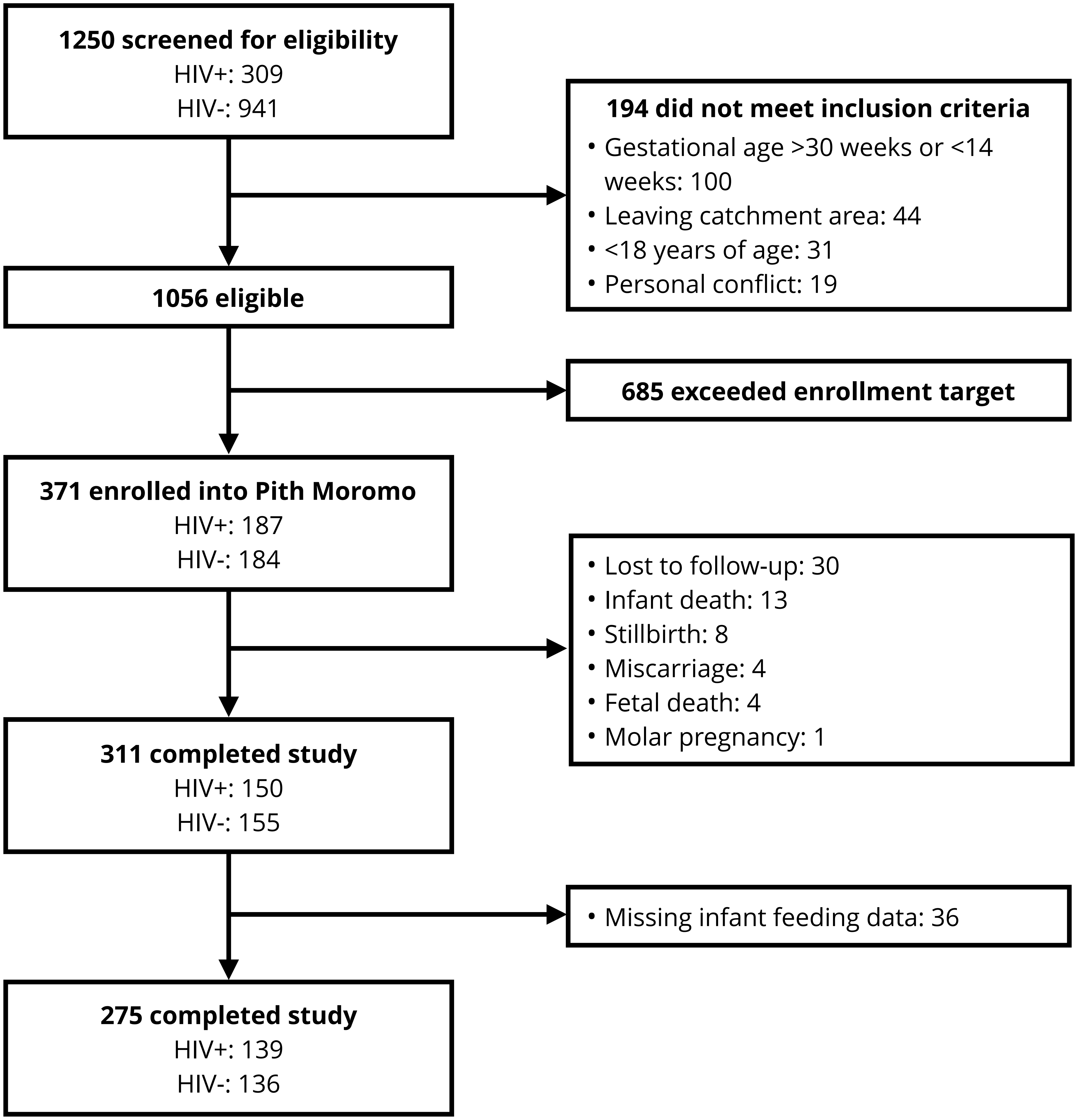

Supplementary Figure 1. Progress of participants through the Pith Moromo study. Three hundred seventy-one women enrolled into the parent study; 8.1% were lost to follow-up.
