# Supplementary material for: HIV infection, hunger, breastfeeding self-efficacy, and depressive symptoms are associated with exclusive breastfeeding to six months among women in western Kenya: a longitudinal observational study
Source: Int Breastfeed J. 2020 Jan 16;15:4. doi: 10.1186/s13006-019-0251-8 (PMC6966845; doi:10.1186/s13006-019-0251-8)
Supplement: Supplementary file 3 — Additional file 3: Figure S3. PM_EBF_Supp_Figure3.pdf; survival curve; Proportion of women (n = 275) exclusively breastfeeding through nine months postpartum, by maternal HIV status. Being HIV-positive was associated with a 64.0% decrease in the likelihood of early exclusive breastfeeding cessation to six months. [file 13006_2019_251_MOESM3_ESM.pdf]

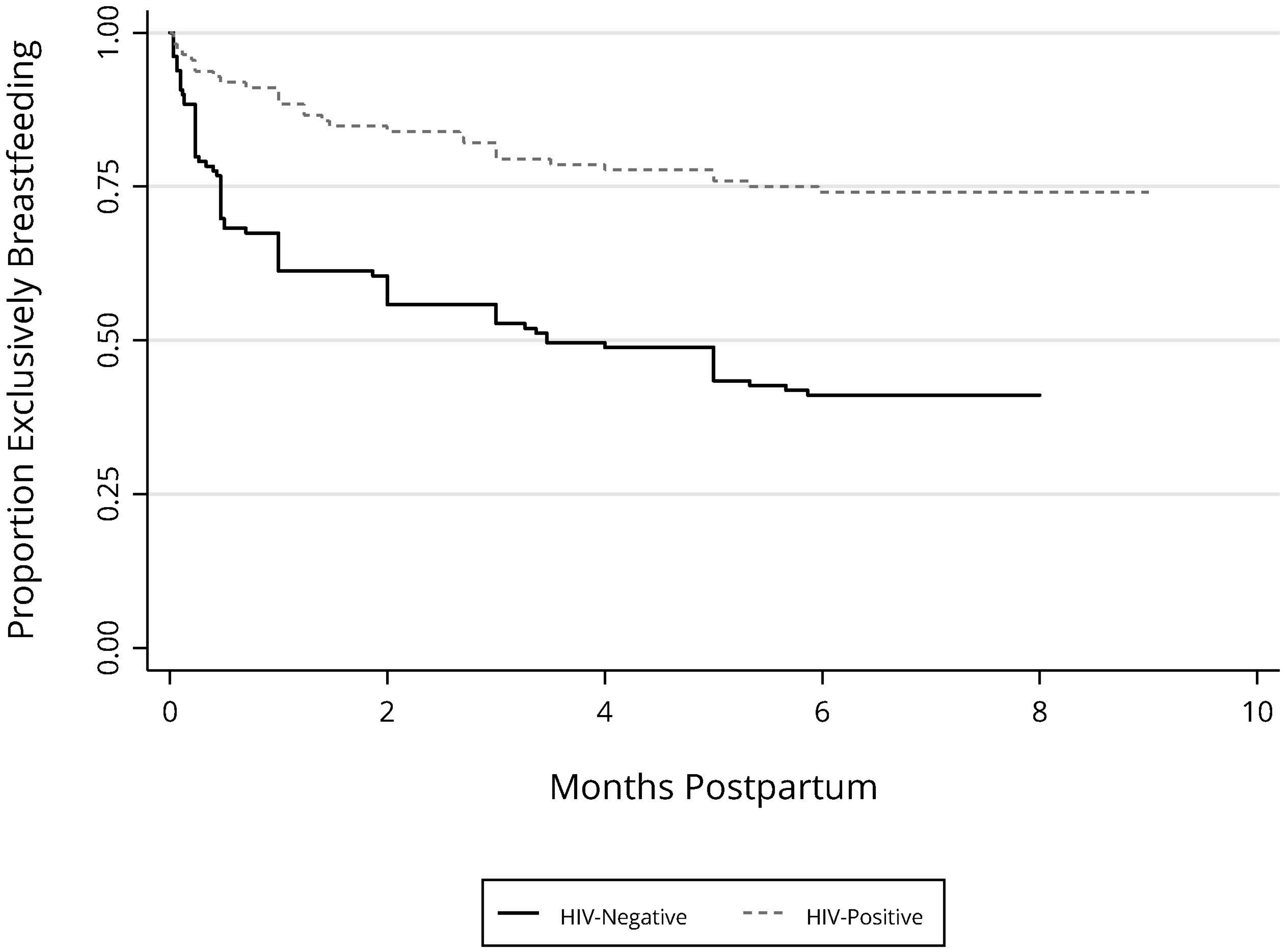

Supplementary Figure 3. Proportion of women (n=275) exclusively breastfeeding through 9 months postpartum, by maternal HIV status. Being HIV-positive was associated with a 64.0% decrease in the likelihood of early exclusive breastfeeding cessation to 6 months .
