# Supplementary material for: HIV infection, hunger, breastfeeding self-efficacy, and depressive symptoms are associated with exclusive breastfeeding to six months among women in western Kenya: a longitudinal observational study
Source: Int Breastfeed J. 2020 Jan 16;15:4. doi: 10.1186/s13006-019-0251-8 (PMC6966845; doi:10.1186/s13006-019-0251-8)
Supplement: Supplementary file 4 — Additional file 4: Table S1. PM_EBF_Supp_Table1.pdf; sensitivity analyses; Bivariate relationship between variables of interest and EBF duration, by cut-off used for determining EBF; only p-values reported for exploratory analysis. [file 13006_2019_251_MOESM4_ESM.pdf]

Supplementary Table 1. Bivariate relationship between variables of interest and EBF duration, by cut-off used for determining EBF; only *p*-values reported for exploratory analysis.

	**5.5 months (53.5% EBF)**	**6 months (52.3% EBF)**
Household characteristics		
Household size	0.908	0.967
Household wealth	0.422	0.445
Maternal characteristics		
Age	0.246	0.315
Ethnic group	0.844	0.933
Religion	0.308	0.386
Relationship status	0.733	0.974
Education level	0.113	0.046
HIV status	<0.001	<0.001
Gravidity	0.904	0.779
Parity	0.733	0.635
Miscarriage	0.751	0.687
Dietary diversity score	0.532	0.512
Individual food insecurity	0.551	0.425
Hunger score	0.249	0.181
Any hunger	0.320	0.248
Social support	0.257	0.317
Perceived stress	0.235	0.186
CES-D depression score	0.110	0.072
Likely depression	0.086	0.064
Quality of life score	0.008	0.003
BMI	0.034	0.028
Fat mass	0.271	0.251
Breastfeeding knowledge	0.956	0.6515
Breastfeeding intent	0.032	0.023
Breastfeeding self-efficacy	0.119	0.202
Breastfeeding social support	0.072	0.155
Infant characteristics		
Infant birthweight	1.000	0.897
Infant sex	0.328	0.281
